# Proposing Novel Data Analytics Method for Anatomical Landmark Identification from Endoscopic Video Frames

**DOI:** 10.1155/2022/8151177

**Published:** 2022-02-23

**Authors:** Shima Ayyoubi Nezhad, Toktam Khatibi, Masoudreza Sohrabi

**Affiliations:** ^1^School of Industrial and Systems Engineering, Tarbiat Modares University (TMU), Tehran, Iran; ^2^Gastrointestinal and Liver Diseases Research Center, Iran University of Medical Sciences (IUMS), Tehran, Iran

## Abstract

**Background:**

The anatomical landmarks contain the characteristics that are used to guide the gastroenterologists during the endoscopy. The expert can also ensure the completion of examination with the help of the anatomical landmarks. Automatic detection of anatomical landmarks in endoscopic video frames can be helpful for guiding the physicians during screening the gastrointestinal tract (GI).

**Method:**

This study presents an automatic novel method for anatomical landmark detection of GI tract from endoscopic video frames based on semisupervised deep convolutional neural network (CNN) and compares the results with supervised CNN model. We consider the anatomical landmarks from Kvasir dataset that includes 500 images for each class of Z-line, pylorus, and cecum. The resolution of these images varies from 750 × 576 up to 1920 × 1072 pixels.

**Result:**

Experimental results show that the supervised CNN has highly desirable performance with accuracy of 100%. Also, our proposed semisupervised CNN can compete with a slight difference similar to the CNN model. Our proposed semisupervised model trained using 1, 5, 10, and 20 percent of training data records as labeled training dataset has the average accuracy of 83%, 98%, 99%, and 99%, respectively.

**Conclusion:**

The main advantage of our proposed method is achieving the high accuracy with small amount of labeled data without spending time for labeling more data. The strength of our proposed method saves the required labor, cost, and time for data labeling.

## 1. Introduction

According to the World Health Organization (WHO), in 2018, stomach and colorectal cancer was among the 5 most common cancers in the world. Altogether, stomach and colorectal cancer accounted for about 2.8 million new cases and 1.6 million deaths in 2018 [1].

According to the development of minimally invasive surgeries (MIS), endoscopy is used to examine the upper gastrointestinal tract (GI), including the esophagus, stomach, and the first part of the small bowel [2].

The anatomical landmarks contain the characteristics that are used to guide the gastroenterologists during the endoscopy [3]. The expert can also ensure the completion of examination with the help of the anatomical landmarks [3]. They are of necessity as a guideline to describe the location of a lesion [3]. Landmarks in the upper GI tract include Z-line and pylorus. Also, one of the landmarks in the lower GI tract is cecum [4]. Z-line is also known as the squamocolumnar junction (SCJ) is a place that the squamous mucosa of the esophagus transitions to the columnar mucosa of the stomach [5, 6]. It works like a border between the esophagus and the stomach. Examination of the Z-line is very useful for measuring the gastric mucosal fold and illustrating sign of reflux [6]. The pylorus is also known as a muscular valve that is around the stomach and the duodenal bulb (or the first part of the small bowel). Both sides of the pylorus must be examined to detect abnormalities like ulcer or erosion [5, 7]. With screening GI tract, the physician can ensure that the pylorus can control the motion of food by condensing muscles [8].

Automatic detection of anatomical landmarks from numerous endoscopic video frames is a main prerequisite task for many endoscopic video analysis applications [9]. For example, detecting and localizing the anatomical landmarks automatically can be helpful to improve the accuracy and speed of physicians in classifying the landmarks [9]. Moreover, diagnosis of anatomical landmarks can be used for following the guidelines that are necessary for screening the GI tract [3]. The report of the physician should include a brief description of anatomical landmarks and image documentation of them [7].

Automatic detection of anatomical landmark has been considered in many previous studies [4, 8, 10].

In the previous studies, the lack of training sample makes the models prone to overfitting and some data would be misclassified. In this study, we try to overcome this problem by proposing semisupervised deep neural networks.

The main objective of this study is proposing an automatic method for landmark detection from the endoscopic video frames. For this purpose, the Kvasir dataset is analyzed in this study.

The main differences of our proposed approach compared with the previous studies which have been analyzed in Kvasir dataset are proposing a semisupervised deep model to reduce the required labeled video frames.

The main novelties of our study and method lie in several folds including the following:

- Proposing a novel method for anatomical landmarks detection from endoscopic video frames
- Proposing a novel semisupervised CNN to overcome the lack of labeled data
- Designing the semisupervised convolutional neural network (SSCNN) on Kvasir dataset
- Comparing the experimental results of supervised and semisupervised CNNs for anatomical landmark detection on Kvasir dataset

This paper is organized as follows. In Section 2, the related works are reviewed.  Section 3 is for describing the dataset and the main step of research methodology in this study. The evaluation of performance metrics and showing how our proposed method works are presented in Section 4. And Section 5 concludes and gives a view for future work.

## 2. Related Works

This section is divided into two folds. At first, the previous studies related to the image processing on endoscopic video frames are considered. Since our aim in this study is to present a semisupervised learning method for classifying the endoscopic video frames, the summary of semisupervised methods is presented in the second fold. More details of each fold will be described in the following sections.

### 2.1. Previous Studies Related to Endoscopic Video Frame Processing

Previous studies focusing on endoscopic video analytics can be divided into methods relying on conventional machine learning methods and deep neural networks [11]. Conventional machine learning methods have extracted handcrafted features from the video frames and then have classified them based on the corresponding extracted feature vectors [12–14]. Deep neural networks can be used as the feature extractor and/or end-to-end classifiers without requiring prior feature extraction from video frames [10, 15, 16].

#### 2.1.1. Previous Methods Relying on Conventional Machine Learning Methods

One of the first researches that has used image processing techniques in endoscopic video frames proposed edge detection methods to find ulcer on GI tract in 1988 [12].

Different previous studies have used image processing techniques for automatic segmentation, classification, detection, and localization of anatomical landmarks and/or diseases [4, 11].

Some of the diseases in GI tract such as polyp [17], tumor [13], cancer [14], ulcer [12], bleeding [18], and esophagitis [19] have been diagnosed in the previous studies based on automatic image processing techniques. Different methods of feature extraction have been used for this purpose in the related works to detect and classify GI tract abnormalities [11]. Color [20], texture [21], and shape [12] descriptors have been extracted and exploited [2] on the spatial or frequency domain [22]. In recent years, deep neural networks have been used for feature extraction from images [23].

Although there are different researches on image processing, they cannot identify which feature is best for demonstrating abnormalities in endoscopic video frames [11]. Different methods for feature extraction have been proposed, but they have not been generalized [11]. Therefore, end-to-end methods have been introduced and helped to represent images efﬁciently [11].

#### 2.1.2. End-to-End Classification using Deep Neural Networks

Another application of deep neural networks is end-to-end classification of images and video frames without requiring prior feature extraction and heavy image preprocessing activities [11, 24].

A previous study has proposed a framework based on convolutional neural networks (CNN) for classifying images using small amount of data [15]. Another model has been presented consisting of a residual neural network (ResNet) followed by a faster region-based CNN (faster R-CNN) [16]. But the authors have mentioned that their proposed framework has shown some limitations for discriminating some classes from others [16].

One of the recent studies has proposed a pipeline including multitype feature extraction method, feature merging, and selection for automatically diagnosis of abnormalities in GI tract [23].

Previous studies have demonstrated that the classification performance has been reduced when the number of classes is increased [23]. Moreover, different classes which have been mostly similar to each other have been misclassified in the previous studies. A proposed solution in the previous studies to overcome this challenge has been increasing the number of training data records [10]. More researches on endoscopic video frames are summarized in Table 1 presented in Appendix A.

As we realized from the previous studies, the lack of enough training samples makes some images misclassified [10]. The solution to addressing this challenge is increasing the number of training data but accessibility to labeled data needs more time and labor [10]. Also, sometimes it is not possible to use domain expert to assign labels to the images accurately. Semisupervised learning has this advantage that need less much labeled data compared to supervised learning methods [25].

Therefore, in this study, we propose a semisupervised method for anatomical landmark identification from endoscopic video frames. In the next section, we summarize semisupervised method.

### 2.2. Previously Proposed Semisupervised Methods

Inaccessibility to labeled data is very common because the expert must spend a large amount of time to assign labels to data records [25]. Therefore, Semisupervised Learning (SSL), which requires a small percent of data records to be labeled previously, can be helpful [25].

As mentioned earlier, sometimes the lack of labeled data makes good performance not achieved on model [26], so SSL method can solve this problem. If we use semisupervised deep learning, we can use the benefit of them to increase the model performance. There are researches that use these methods [27, 28].

SSL is one of the machine learning approaches that lies between supervised and unsupervised learning [29, 30]. The main advantage of SSL methods is that they require smaller volume of labeled dataset for training the models [29, 30]. They use both labeled and unlabeled data records simultaneously for SSL training phase [29, 30].

There are some important assumptions in SSL. The first assumption is about data distribution smoothness [29, 30]. The second assumption says that the marginal region between two different classes has low density [29, 30]. In the input space with higher dimensions, the data records usually lie on manifolds having lower dimensions with a smooth shape [29, 30]. Finally, the similar data records should have similar class labels [29, 30].

Different previously proposed SSL methods have exploited two different learning modes, including inductive and transductive learning [31]. Inductive learning methods predict the class label of the unlabeled data records which have not been presented to the model during its training [31]. In the concept of transductive learning introduced by Vapnik and Sterin [32], both labeled and unlabeled training data records have been fed to the model during its training [25].

Inductive SSL methods include self-training [33], co-training [34], and Expectation Maximization (EM) with generative mixture model [35]. Recently because of the powerful performance of deep learning models in supervised learning, the SSL methods are focused on them [26]. There are very different architectures of deep learning model like convolutional neural network (CNN) [36], recurrent neural network (RNN) [37], autoencoders [38], and generative adversarial networks (GANs) [39].

Semisupervised Support Vector Machines (S3VMs) [32] and graph-based method [40] are examples of transductive SSL methods.

Inductive methods make a classifier model cover the entire input space but transductive methods do not [30]. The prediction abilities of the transductive methods have been limited to the training samples and have been prone to overfitting and loss of generalization ability [30].

Therefore, inductive methods are used in this study to make a classifier that can classify any object in input space with high accuracy and not limited to the data that has been seen in training phase [30].

A previous study has proposed a semisupervised convolutional neural network (SSCNN) model with an iterative manner in which the labeled data and unlabeled data that have high prediction confidence score in the previous iteration have been used as the training sample for the next iteration [26].

Another research has used active learning to find the reliable data from unlabeled data to add into training data set and then has developed semisupervised methods by adding a novel term into loss function of CNN [41].

In [42], the researchers have designed and used a graph-based SSL method to learn the class label of unlabeled data records. Moreover, for overcoming the model overfitting, the data augmentation using GANs has been performed to enrich the training dataset.

A previous study has designed an ensemble model to combine the results of the feedforward designed convolutional neural networks (FF-CNNs) to improve the performance of SSL learning [43].

Another SSCNN model has been designed and proposed in a previous study to extract the features and classify the images. The network determines the probability of each class by using a Soft-max activation function in the output layer [44].

## 3. Materials and Methods

**Figure 1:**
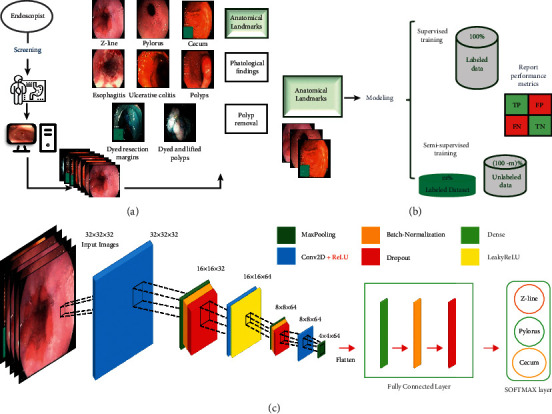
The main steps of the proposed method for anatomical landmark detection from the endoscopic video frames. (a) Kvasir dataset. (b) The main step of our research methodology. (c) The architecture of CNN.

This section is divided into three folds. At first, we introduce the details of Kvasir dataset. The second fold describes our designed and proposed semisupervised method. Finally, evaluation metrics are presented. More details about each fold are described in the next sections. We use the Cross-Industry Process for Data Mining tasks (CRISP-DM) methodology for designing our research method as shown in Figure 1 [45]. CRISP-DM is a standard framework for data mining projects introduced by Wirth and Hipp for designing the process of data mining problem [45].

### 3.1. Dataset Description

In this section, we first introduce our analyzed dataset of annotated endoscopic video frames. The dataset used in this study is Kvasir dataset that includes 4000 images captured from inside the GI tract [7]. The video frames of Kvasir are classified into 8 classes based on anatomical landmark and pathological findings. The classes are esophagitis, polyps and ulcerative colitis, and polyp removal including the dyed and lifted polyp and the dyed resection margins [7]. In this study, the anatomical landmark images that are analyzed include 500 images for each class of Z-line, pylorus, and cecum [7]. The resolution of these images varies from 750 × 576 up to 1920 × 1072 pixels [7].  Figure 1(a) illustrates different classes of video frames in Kvasir dataset.

### 3.2. Our Proposed Classification Method

Our proposed method, as shown in Figure 1, consists of two different classification methods based on supervised and semisupervised learning. More details about each method are described in the following sections.

#### 3.2.1. Supervised Learning

At first, we design and propose a supervised end-to-end CNN trained based on all training dataset considering their class labels. CNNs have been applied to solve different problems in machine learning [46]. The important advantage of using CNNs is that they can learn hierarchical local and global features from high-dimensional raw data without needing any prior method for segmentation and/or feature extraction from the data [47]. More details about CNNs are explained in Appendix B.

Before designing CNNs, the data is partitioned into original training and test datasets with a ratio of 80 : 20. Then, the original training dataset is partitioned into training and validation subsets with a ratio of 75 : 25. Training subset is used for training the classifier and the validation subset is used for tuning the hyperparameters of the model to address issues such as overfitting. For this purpose, grid search method is used for tuning the hyperparameters. Then, the performance is evaluated by applying the classifier into the validation subset to choose the best combinations of the hyperparameters' values.

Different architectures for CNN are examined and the architecture that has the best performance for training and validation subsets is selected as shown in Figure 1(c).

Table 1 shows the architecture of CNN model for anatomical landmark detection from endoscopic video frames.

CNNs are trained for 60 epochs with Adam optimizer with learning rate of 0.001 and batch size of 8. The activation function for all layers except last layer is ReLU. The last layer uses Soft-Max.

#### 3.2.2. Semisupervised Learning

**Figure 2:**
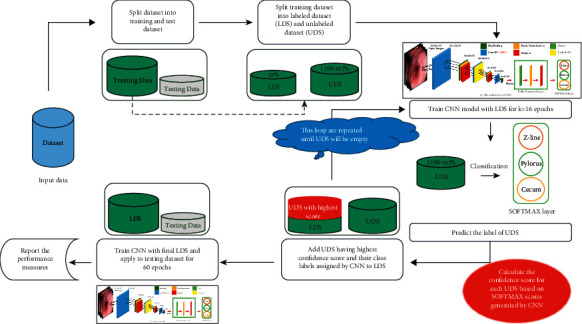
The architecture of our proposed and designed semisupervised convolutional neural network (SSCNN).

We use SSL methods in this study to overcome the lack of training data. The architecture of our proposed and designed SSL method is illustrated in Figure 2.

As shown in Figure 2, the main steps of our proposed and designed SSCNN are described in Algorithm 1.

The main step for calculating the confidence score is explained in Algorithm 2.

### 3.3. Evaluation Metrics

The performance of the model can be evaluated by performance metrics like accuracy, precision, recall, *F*1-score, and Area under Receiver Operating Characteristics (ROC) curve (AUC) [48].

The value of accuracy shows the classiﬁer's predictive abilities as follows [48]:

(1) $$\begin{array}{c} \text{Accuracy}=\frac{\text{TP}+\text{TN}}{N}, \end{array}$$

where TP is abbreviation of true positives, TN is abbreviation of true negatives, and *N* is the all number of data records.

Precision denotes how many data assigned the positive label by the model and the real class label is positive [48]. This measure is calculated as follows:

(2) $$\begin{array}{c} \text{Precision}=\frac{\text{TP}}{\text{TP}+\text{FP}}. \end{array}$$

Recall is also known as *true positive rate* denoted in equation (3) and it shows that the ratio of samples is correctly identified as positive class.

(3) $$\begin{array}{c} \text{Recall}=\frac{\text{TP}}{\text{TP}+\text{FN}}, \end{array}$$

where FP is abbreviation of false positives and FN is abbreviation of false negatives.

The *F*1-measure is the harmonic mean of precision and recall, as show in the following equation [48]:

(4) $$\begin{array}{c} F1-\text{measure}=2\times \frac{\text{precision}\times \text{recall}}{\text{precision}+\text{recall}}. \end{array}$$

Some of these measures are suitable for binary classification but for multiclass classification; the measure performances are calculated as equations (5)–(10) [49]:

(5) $$\begin{array}{c} \text{micro}-\text{averaged precision}=\frac{\sum_{c=1}^{NOC}{\text{TP}}_{c}}{\sum_{c=1}^{NOC}{\text{TP}}_{c}+\sum_{c=1}^{NOC}{\text{FP}}_{c}}, \end{array}$$

(6) $$\begin{array}{c} \text{micro}-\text{averaged recall}=\frac{\sum_{c=1}^{NOC}{\text{TP}}_{c}}{\sum_{c=1}^{NOC}{\text{TP}}_{c}+\sum_{c=1}^{NOC}{\text{FN}}_{c}\;}, \end{array}$$

(7) $$\begin{array}{c} \text{micro}-\text{averaged }F1-\text{score}=2\times \frac{\text{micro}-\text{averaged precision}\times \text{micro}-\text{averaged precision}}{\text{micro}-\text{averaged precision}+\text{micro}-\text{averaged precision}},\; \end{array}$$

(8) $$\begin{array}{c} \text{macro}-\text{averaged precision}=\frac{1}{NOC}\times \sum_{c=1}^{NOC}\frac{{\text{TP}}_{c}}{{\text{TP}}_{c}+{\text{FP}}_{c}}, \end{array}$$

(9) $$\begin{array}{c} \text{macro}-\text{averaged recall}=\frac{1}{NOC}\times \sum_{c=1}^{NOC}\frac{{\text{TP}}_{c}}{{\text{TP}}_{c}+{\text{FN}}_{c}}, \end{array}$$

(10) $$\begin{array}{c} macro-average\;d\;F1-score=\;\frac{1}{NOC}\times \sum_{c=1}^{NOC}2\times \frac{\left({\text{TP}}_{c}/{\text{TP}}_{c}+{\text{FP}}_{c}\right)\times \left({\text{TP}}_{c}/{\text{TP}}_{c}+{\text{FN}}_{c}\right)\;}{\left({\text{TP}}_{c}/{\text{TP}}_{c}+{\text{FP}}_{c}\right)+\left({\text{TP}}_{c}/{\text{TP}}_{c}+{\text{FN}}_{c}\right)\;}. \end{array}$$

In the above equations, NOC is the number of different classes.

## 4. Results and Discussion

In this section, the performance measures of each proposed model are reported to know which model can better identify and classify the anatomical landmarks.

SSCNN model is trained for 1, 5, 10, and 20 percent of labeled data and the performance metrics are reported in Table 2. Table 2 illustrates the average of the performance measures for each model for anatomical landmarks identification from endoscopic video frames.

As shown in Table 2, the best performance belongs to the supervised CNN model. Our aim is to find the best performance in SSCNN model, which can compete with the supervised CNN.

Results listed in Table 2 show that training the SSCNN model with small amount of labeled data has acceptable performances like supervised CNN model, but the performance of the last SSCNN model which is trained with 1 percent of labeled data, is decreased.

Table 3 indicates the macro performance measures of the proposed model for anatomical landmarks detection from endoscopic video frames separately for each class.

As illustrated in Table 3, supervised CNN has the best performance to detect each class. On the other hand, the proposed SSCNN models except the last one, which is trained by 1 percent of labeled data, have acceptable performances.

**Figure 3:**
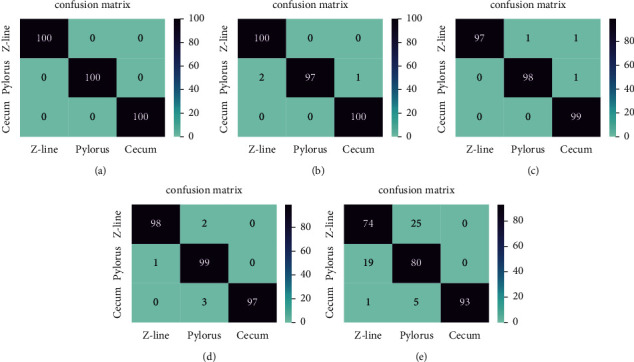
The confusion matrix for different model. (a) Supervised CNN, (b) SSCNN (*m* = 20%), (c) SSCNN (*m* = 10%), (d) SSCNN (*m* = 5%), and (e) SSCNN (*m* = 1%).

Figure 3 demonstrates the confusion matrix of each model. As depicted in Figure 3, the supervised CNN model classifies anatomical landmarks correctly. In the confusion matrix of SSCNN which is trained by 20 percent of labeled data, only 3 video frames out of 300 are classified wrongly. In the SSCNN model which is trained by 10 percent of labeled data, only 3 video frames out of 297 are misclassified. In the SSCNN model, that 5 percent of labeled data participates in training the model; 6 video frames out of 300 are misclassified. But in the last model, misclassified video frames are increased to 50 video frames out of 297.

**Figure 4:**
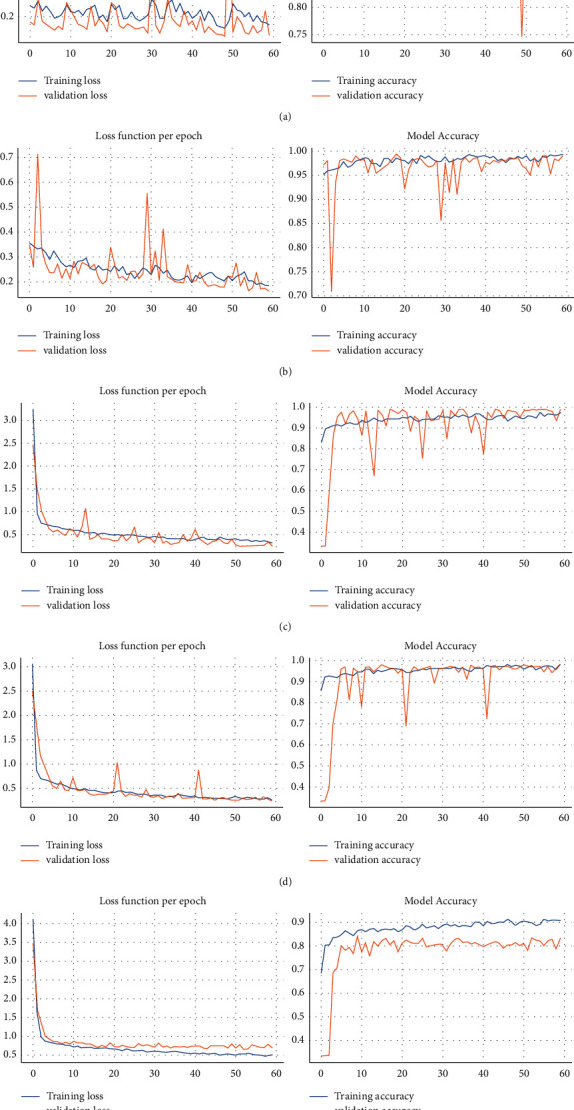
Training and validation curves (accuracy and loss function per epoch). (a) Supervised CNN, (b) SSCNN (*m* = 20%), (c) SSCNN (*m* = 10%), (d) SSCNN (*m* = 5%), and (e) SSCNN (*m* = 1%).

Figure 4 illustrates the accuracy and loss functions per epochs for each model. As shown in Figure 4, the accuracy and loss functions of each model except the last model for training and test dataset, overfitting has not occurred during training the models. As depicted in Figure 4(d), the last model is at the risk of overfitting.

**Figure 5:**
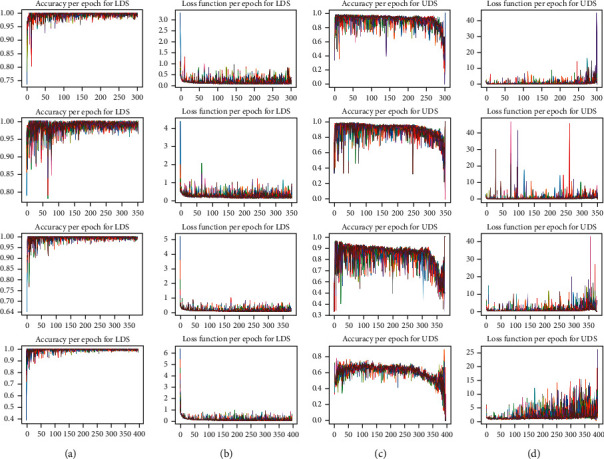
The accuracy and loss function per epoch for LDS and UDS during the train SSCNN models. (a) SSCNN (*m* = 20%), (b) SSCNN (*m* = 10%), (c) SSCNN (*m* = 5%), and (d) SSCNN (*m* = 1%).

Figure 5 shows the accuracy and loss function of LDS and UDS during training the SSCNN models. At each step, only one data of each class that has highest confidence score in their class is added to the LDS. So, the different colors in Figure 5 depict the number of epochs that SSCNN model is run to discharge the UDS.

**Figure 6:**
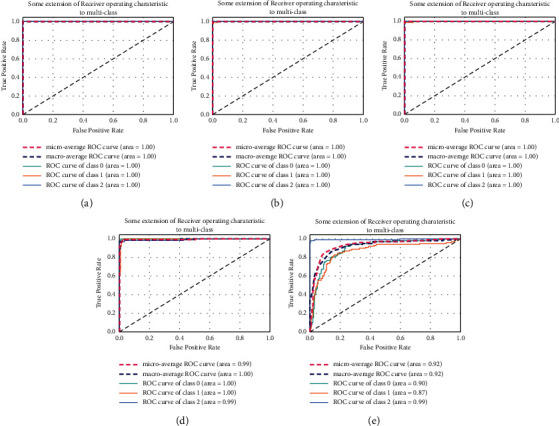
The ROC curve. (a) Supervised CNN, (b) SSCNN (*m* = 20%), (c) SSCNN (*m* = 10%), (d) SSCNN (*m* = 5%), and (e) SSCNN (*m* = 1%).

Figure 6 demonstrates the ROC curve of each model. As illustrated in Figure 5, the AUC of each model except the last one is highly desirable.

**Figure 7:**
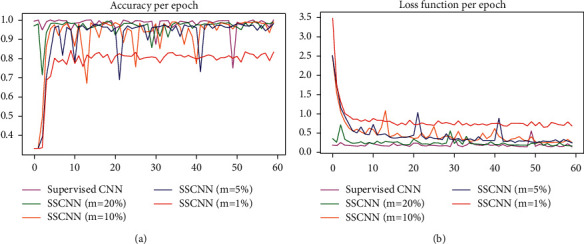
The accuracy and loss function of each models per epoch. (a) Accuracy per epoch. (b) Loss function per epoch.

To compare supervised CNN model with our proposed models, Figure 7 demonstrates the accuracy and loss function per epoch of each model.

Table 4 indicates the processing time details for each model in this study, which is calculated by “Google Colab.” In this platform, the maximum amount of RAM is 12.76 GB and the maximum amount of disk is 68.40 GB, which is allocated to users. The GPU models that can be used in “Google Colab” are NVIDIA K80, P100, P4, T4, and V100 GPUs.

The main aim of this study is proposing the semisupervised model that has good performance to detect the anatomical landmarks from endoscopic video frames. Our proposed model has the best performance and its performance is acceptable against the supervised model.

The use of SSL method in this study has caused the training model, which is learned with a small sample of labeled data, can classify the test data with high accuracy. This method is helpful to vanquish the lack of labeled data.

To validate the advantage of the proposed method, we compared it with different state-of-the-art semisupervised learning algorithms on similar dataset in Table 5.

Comparing the performance of the semisupervised learning algorithms in Table 5 , it can be appreciated that our method leads to superior performance especially when the labeled data is insufficient or access to the labeled data is impossible. But our method also has some weakness. Our method just focused on anatomical landmarks, which is included in three different classes.

## 5. Conclusion

The anatomical landmark detection is a very important task to guide the physician during screening the GI tract. In this study, an automatic novel method based on semisupervised learning of deep convolutional neural networks is proposed for anatomical landmark detection of GI tract from the endoscopic video frames on Kvasir dataset. The considered landmarks include Z-line and pylorus in the upper GI tract and cecum in the lower of GI tract.

The main novelty of this study is using both of supervised and semisupervised learning methods together and comparing the results of them. First, the supervised CNN is trained, and the performance measures are reported. Then, the different semisupervised CNNs (SSCNNs) are designed and trained for anatomical landmark detection from endoscopic video frames especially when the labeled data is insufficient. In SSCNNs, data is partitioned into training and test datasets. Then, the training dataset is partitioned into UDS and LDS with ratio of *m*:(100 − *m*). The SSCNNs are trained by LDS and predict UDS data records. UDS data records having the maximum confidence score are added to LDS and excluded from UDS. These steps are repeated until UDS will be empty.

The supervised CNN achieves the best performance in identification of anatomical landmarks. Also, the experimental results of our proposed semisupervised method show high accuracy for anatomical landmark detection. The proposed SSCNN with 1, 5, 10, and 20 percent of training data records included in LDS has the average accuracy of 83%, 98%, 99%, and 99%, respectively. The results demonstrate the desirable performance of our proposed method while it uses the fewer samples of labeled data for training the model. This method saves the required labor, cost, and time for data labeling. SSCNN model which is trained by 1 percent of labeled data is exposed to overfitting while the SSCNN model with 5 percent of labeled data has good performance.

A main limitation of this study is considering two anatomical landmarks from upper GI tract and one of them from lower GI tract while there are eight anatomical landmarks in the upper GI tract and eight anatomical landmarks in the lower GI tract [3]. It is recommended for the future studies to provide and collect datasets considering more anatomical landmarks and demographic features for further analysis.

A potential solution when the labeled data has too fewer records can be using data augmentation methods to improve the performance measures of the model. A future research direction can be using the data augmentation methods in the preprocessing step of this method to improve the accuracy of model, which is trained with lower than 5 percent of labeled data.

## Figures and Tables

**Algorithm 1:**
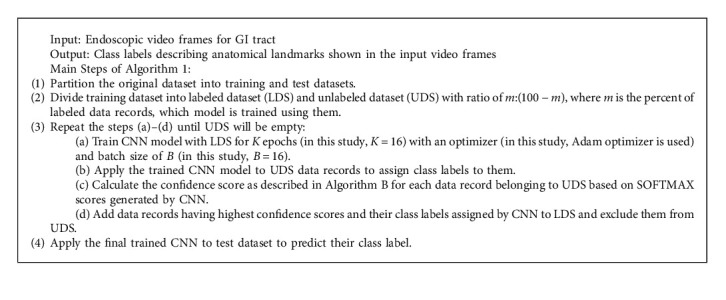
The steps for training SSCNN.

**Algorithm 2:**
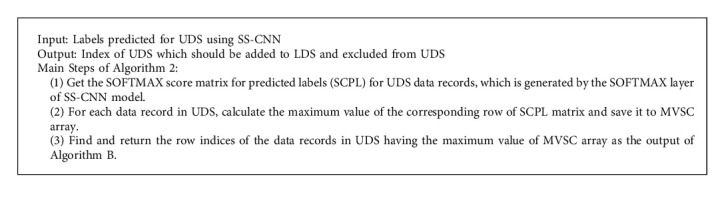
The steps for calculating the confidence score.

**Table 1:** CNNs architecture for anatomical landmark detection from endoscopic video frames.

Layer	Output shape	Parameters
Input layer	(None, 32, 32, 3)	0
Conv2D	(None, 32, 32, 32)	896
MaxPooling	(None, 16, 16, 32)	0
Batch-normalization	(None, 16, 16, 32)	128
Dropout	(None, 16, 16, 32)	0
Conv2D	(None, 16, 16, 64)	18496
LeakyReLU	(None, 16, 16, 64)	0
MaxPooling2D	(None, 8, 8, 64)	0
Batch-normalization	(None, 8, 8, 64)	256
Dropout	(None, 8, 8, 64)	0
Conv2D	(None, 8, 8, 64)	36928
MaxPooling2D	(None, 4, 4, 64)	0
Flatten	(None, 1024)	0
Dense	(None, 128)	131200
Batch-normalization	(None, 128)	512
Dropout	(None, 128)	0
Dense	(None, 3)	387

**Table 2:** The performance measures of the proposed model for anatomical landmark identification from endoscopic video frames.

Performance metrics	Supervised CNN	SSCNN (*m* = 20%)	SSCNN (*m* = 10%)	SSCNN (*m* = 5%)	SSCNN (*m* = 1%)
Accuracy	100.00	99.00	99.00	98.00	83.00
Micro-precision	100.00	99.00	98.98	98.00	83.16
Micro-recall	100.00	99.00	98.98	98.00	83.16
Micro-*F*1-score	100.00	99.00	98.98	99.45	83.16
Micro-AUC	100.00	99.98	99.96	98.00	92.43
Macro-precision	100.00	99.00	99.00	98.00	84.00
Macro-recall	100.00	99.00	99.00	98.00	83.00
Macro-*F*1-score	100.00	99.00	99.00	98.00	83.00
Macro-AUC	100.00	99.98	99.97	99.52	91.91

**Table 3:** The macro performance measure of proposed model for anatomical landmark identification from endoscopic video frames.

Anatomical landmarks	Model	Accuracy	Precision	Recall	*F*1-score	AUC
Z-line	Supervised CNN	100.00	100.00	100.00	100.00	100.00
	SSCNN (*m* = 20%)	99.00	98.00	100.00	99.00	99.99
	SSCNN (*m* = 10%)	99.00	100.00	98.00	99.00	100.00
	SSCNN (*m* = 5%)	98.00	99.00	98.00	98.00	99.97
	SSCNN (*m* = 1%)	83.00	79.00	75.00	77.00	89.80

Pylorus	Supervised CNN	100.00	100.00	100.00	100.00	100.00
	SSCNN (*m* = 20%)	99.00	100.00	97.00	98.00	99.95
	SSCNN (*m* = 10%)	99.00	99.00	99.00	99.00	99.93
	SSCNN (*m* = 5%)	98.00	95.00	99.00	97.00	99.55
	SSCNN (*m* = 1%)	83.00	73.00	81.00	77.00	86.58

Cecum	Supervised CNN	100.00	100.00	100.00	100.00	100.00
	SSCNN (*m* = 20%)	99.00	99.00	100.00	100.00	100.00
	SSCNN (*m* = 10%)	99.00	98.00	100.00	99.00	99.98
	SSCNN (*m* = 5%)	98.00	100.00	97.00	98.00	99.03
	SSCNN (*m* = 1%)	83.00	100.00	94.00	97.00	99.35

**Table 4:** Processing time details for each model.

Model	Execution time for training (seconds)	Execution time for testing (seconds)
Supervised CNN	137.07	48.61
SSCNN (m = 20%)	8616.45	49.02
SSCNN (m = 10%)	10140.54	54.41
SSCNN (m = 5%)	10444.81	58.71
SSCNN (m = 1%)	10701.25	59.16

**Table 5:** Comparing the performance of different state-of-the-art semisupervised learning algorithms on similar dataset.

Author	Year	Dataset	Performance metrics
Wu et al. [50]	2021	Kvasir-SEG and CVC-Clinic DB	Dice coefficient = 80.95
Zhang et al. [51]	2021	ISIC 2017 skin lesions dataset and the Kvasir-SEG polyp dataset	Dice coefficient = 85.10
Inés et al. [52]	2021	Kvasir V2	Accuracy = 93.00
Gjestang et al. [53]	2021	Hyper-Kvasir, and Kvasir-capsule	Accuracy on hyper-Kvasir = 89.30
			Accuracy on Kvasir-capsule = 69.50
Ours (SSCNN “*m* = 20%”)	2021	Kvasir V1 (anatomical landmarks)	Accuracy = 99.00

## Data Availability

The data are publicly available at https://datasets.simula.no/kvasir/ as Kvasir Dataset v1.

## Supplementary Materials

Supplementary Materials
*Appendix A*. Summary of related works. *Table A.1*. A summary of the previous studies for abnormality diagnosis of the endoscopic videos of GI tract. *Appendix B*. More details about CNNs. *Figure B.* SEQ Figure \∗ ARABIC *1*. CNN architecture. *Figure B.* SEQ Figure \∗ ARABIC *2*. A sample of convolution operation in CNN. *Figure B.* SEQ Figure \∗ ARABIC *3*. A sample of max-pooling operation in CNN.
