# Supplementary material for: Proposing Novel Data Analytics Method for Anatomical Landmark Identification from Endoscopic Video Frames
Source: J Healthc Eng. 2022 Feb 23;2022:8151177. doi: 10.1155/2022/8151177 (PMC8890842; doi:10.1155/2022/8151177)
Supplement: Supplementary Materials — Appendix A. Summary of related works. Table A.1. A summary of the previous studies for abnormality diagnosis of the endoscopic videos of GI tract. Appendix B. More details about CNNs. Figure B. SEQ Figure \∗ ARABIC 1. CNN architecture. Figure B. SEQ Figure \∗ ARABIC 2. A sample of convolution operation in CNN. Figure B. SEQ Figure \∗ ARABIC 3. A sample of max-pooling operation in CNN. [file 8151177.f1.zip › 8151177.f1/supplementaryMaterials_AppendixA (2).docx]

**Appendix A Summary of related works**

**Table A.1** A Summary of the previous studies for abnormality diagnosis the endoscopic videos of GI tract

| Performance | Dataset | Technique | Category | Features | Refs. | Year |
| --- | --- | --- | --- | --- | --- | --- |
| Accuracy = 93% | 4000 images from Kvasir dataset [[7](#_ENREF_7)] | CNN | Classification GI diseases and anatomical landmarks | Feature extraction using CNNs | [[15](#_ENREF_15)] | 2017 |
| MCC^[[1]](#footnote-1)^ = 94.24% | 4900 bounding boxes | ResNet and Faster R-CNN | abnormality finding and anatomical landmark detection | Feature extraction using CNNs | [[16](#_ENREF_16)] | 2019 |
| Accuracy = 91.5% | 4000 images from Kvasir dataset [[7](#_ENREF_7)] | CNN | Detection of GI Diseases | Feature extraction using CNNs | [[10](#_ENREF_10)] | 2017 |
| Accuracy = 98.48% | 4000 images from Kvasir dataset [[7](#_ENREF_7)] | Inception-v4, Inception-ResNet-v2, and NASNet^[[2]](#footnote-2)^ | Classification GI diseases and anatomical landmark | Feature extraction using CNNs | [[4](#_ENREF_4)] | 2019 |
| MCC = 90.2% | 110,079 images and 374 videos | Averaged ResNet-152 + DenseNet-161 | Classification GI diseases and anatomical landmark | Feature extraction using CNNs | [[9](#_ENREF_9)] | 2020 |
| Sensitivity = 90% , specificity = 70% | 49 nonneoplastic,160 neoplastic polyps | SVM^[[3]](#footnote-3)^ | Polyp classification in 2 classes | Features based on characteristics of blood vessels | [[17](#_ENREF_17)] | 2009 |
| Sensitivity = 97.8% , specificity = 96.7% | 900 normal images, 900 typical tumor images | SVM | Automatic diagnosis and classification tumor | Texture descriptors | [[13](#_ENREF_13)] | 2016 |
| Accuracy = 91% | 176 images | LMT^[[4]](#footnote-4)^, Naive-Bayes, IB1^[[5]](#footnote-5)^ and SMO^[[6]](#footnote-6)^ | Cancer detection and classification | Color and texture | [[14](#_ENREF_14)] | 2009 |
| Process the image for 4 to 9 minutes | Images with size of 256*256 | - | Ulcer detection | Edge detection and color | [[12](#_ENREF_12)] | 1988 |
| Accuracy = 97.6% | 300 bleeding frames and 200 nonbleeding frames | SVM | Bleeding detection and classification | Color and texture | [[18](#_ENREF_18)] | 2019 |
| Sensitivity = 90% | 1200 frames | - | Barrett's esophagus detection | Color | [[19](#_ENREF_19)] | 2001 |
| Accuracy = 99.12% | 1251 images | BPNN^[[7]](#footnote-7)^ | Classification capsule endoscopy images (CEIs) | Color and texture | [[20](#_ENREF_20)] | 2019 |
| Accuracy = 93.16% in telangiectasia lesions detection | 344 images | SVM | Abnormalities detection of small bowel | Texture | [[21](#_ENREF_21)] | 2015 |
| AUC = 0.956 | 4000 images from Kvasir dataset [[7](#_ENREF_7)] | SVM- CNN-Naïve Bayes and RF^[[8]](#footnote-8)^ | Classifying 8 different classes based on anatomical landmarks, pathological findings and endoscopic procedures | Texture and color | [[2](#_ENREF_2)] | 2017 |
| Accuracy = 99.13% | 32 videos | SVM | Bleeding detection and classification | Texture descriptors in the frequency domain | [[22](#_ENREF_22)] | 2015 |
| Accuracy = 96.5% | 9889 images | SVM- EBT^[[9]](#footnote-9)^ and ESKNN-^[[10]](#footnote-10)^  KNN | Classification of gastric diseases | Feature extraction using VGG16^[[11]](#footnote-11)^ | [[23](#_ENREF_23)] | 2020 |

1. Matthews correlation coefficient: MCC [↑](#footnote-ref-1)
2. Neural architecture search net: NASNET [↑](#footnote-ref-2)
3. Support vector machine: SVM [↑](#footnote-ref-3)
4. Logistic model trees: LMT [↑](#footnote-ref-4)
5. K=1 -nearest neighbors: IB1 [↑](#footnote-ref-5)
6. Sequential minimal optimization: SMO [↑](#footnote-ref-6)
7. Back propagation neural network: BPNN [↑](#footnote-ref-7)
8. Random forest: RF [↑](#footnote-ref-8)
9. Ensemble bagged tree: EBT [↑](#footnote-ref-9)
10. Ensemble subspace K-nearest neighbors: ESKNN [↑](#footnote-ref-10)
11. Visual geometry group: VGG [↑](#footnote-ref-11)
