# Supplementary material for: Proposing Novel Data Analytics Method for Anatomical Landmark Identification from Endoscopic Video Frames
Source: J Healthc Eng. 2022 Feb 23;2022:8151177. doi: 10.1155/2022/8151177 (PMC8890842; doi:10.1155/2022/8151177)
Supplement: Supplementary Materials — Appendix A. Summary of related works. Table A.1. A summary of the previous studies for abnormality diagnosis of the endoscopic videos of GI tract. Appendix B. More details about CNNs. Figure B. SEQ Figure \∗ ARABIC 1. CNN architecture. Figure B. SEQ Figure \∗ ARABIC 2. A sample of convolution operation in CNN. Figure B. SEQ Figure \∗ ARABIC 3. A sample of max-pooling operation in CNN. [file 8151177.f1.zip › 8151177.f1/supplementaryMaterials_AppendixB (2).docx]

**Appendix B: More detail about CNNs**

CNNs are a kind of traditional deep neural networks which are made up by multi layers of neurons but CNNs have lower in term of memory and computation time than the traditional model [[50](#_ENREF_50)]. CNNs consist of several convolutional block that includes convolutional layers and pooling or down-sampling layers and dense or fully connected (FC) layers [[46](#_ENREF_46)]. CNNs architecture is shown in Figure. B.1.

**
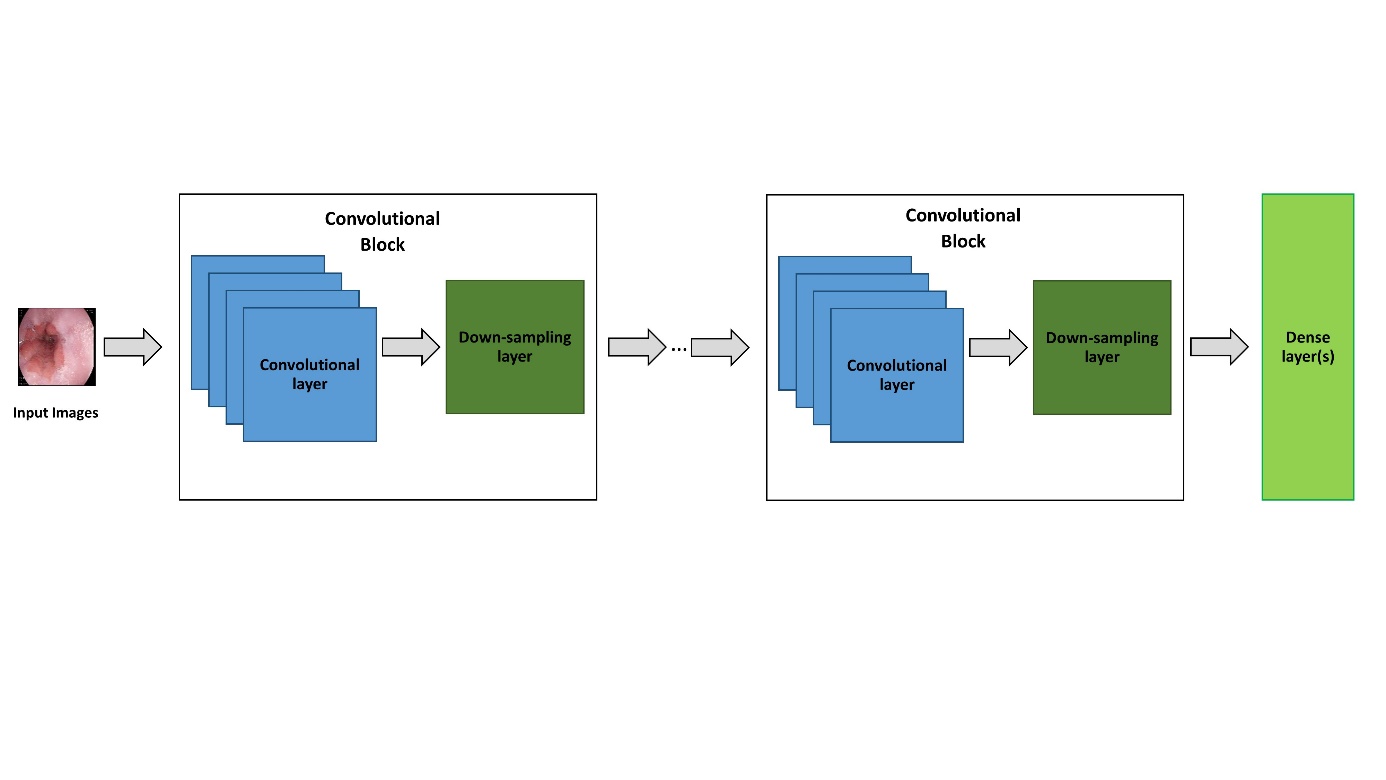
**

**Figure B.1** CNN architecture

Convolutional blocks are used as feature learning component and dense layers are used as classification component [[46](#_ENREF_46)].

A sample of convolutional operation is shown in Figure. B.2. Feature map is produced by convolutional layer [[50](#_ENREF_50)]. The convolutional layer consists of filters, and the convolutional operation between filters and the input data creates feature maps [[50](#_ENREF_50)].


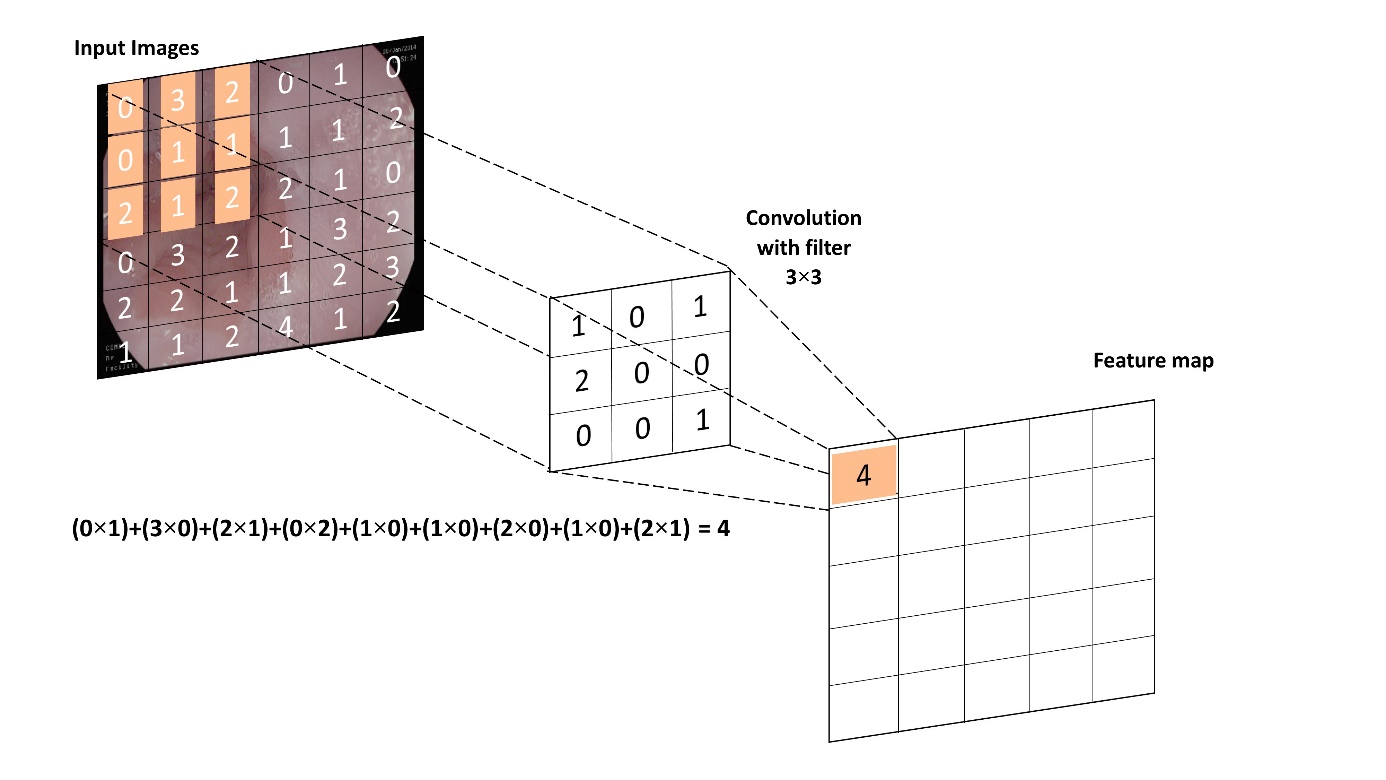
**Figure B.2** A sample of convolution operation in CNN

The aim of the down-sampling layer is to decrease the spatial size of the feature map which produced by convolutional layer [[50](#_ENREF_50)].

A sample of dawn-sampling operation with max-pooling is shown in Figure. B.3.

**
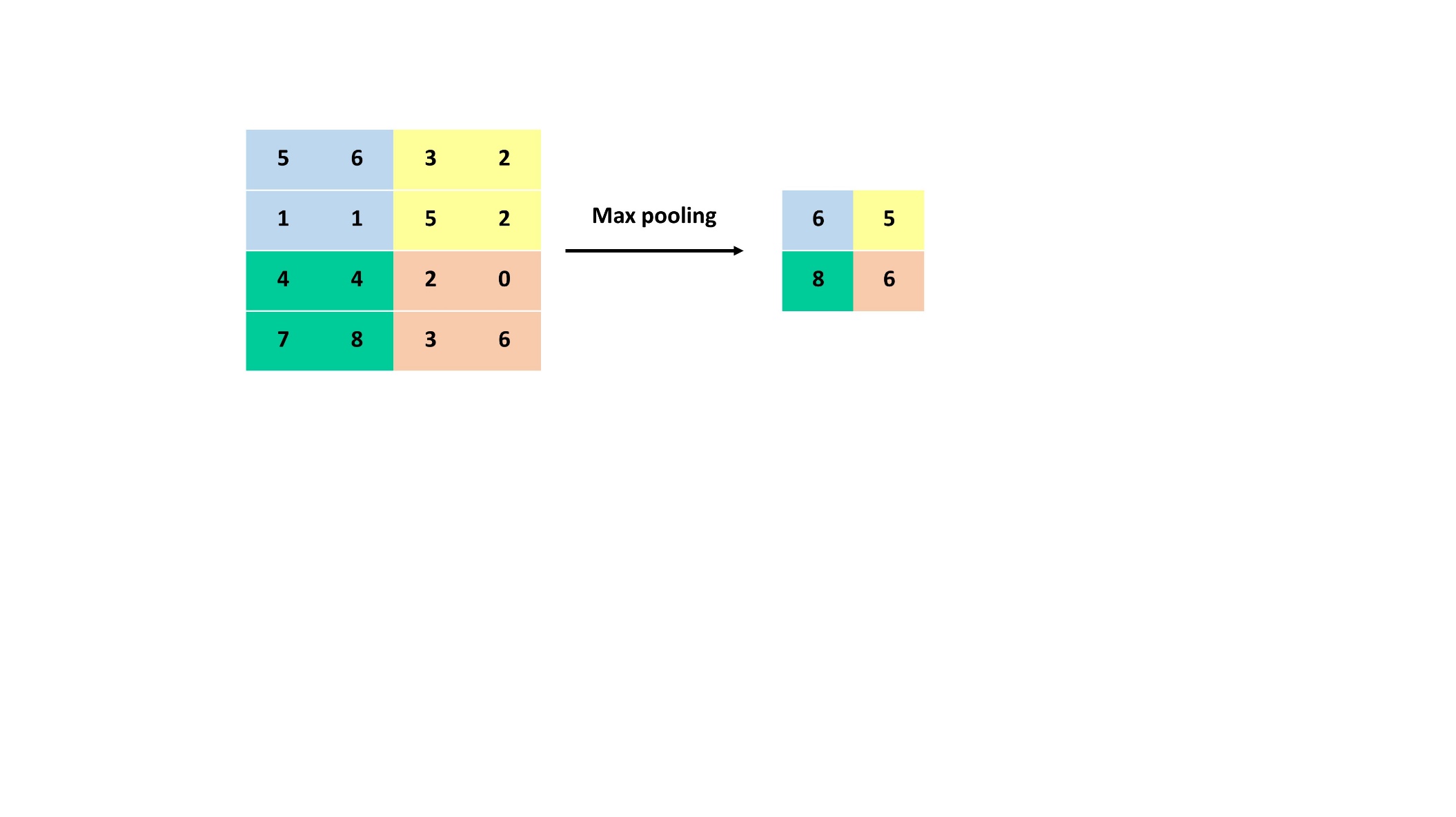
**

**Figure B.3** A sample of max-pooling operation in CNN

The last layers of CNN are FC layers. The function of these layers are similar to Deep neural network. These layers are classified the features which are extracted by the convolutional blocks [[50](#_ENREF_50)]. The feature maps inputted in the FC or dense layers are flatted into 1D vector [[46](#_ENREF_46)].

In CNNs, after the computation of the convolutional layer's operation used nonlinear activation functions [[50](#_ENREF_50)]. The Rectified Linear Unit (ReLU) function is common. This function has good performance to overcome vanishing and exploding gradient [[51](#_ENREF_51)]. ReLU is shown in Eq. (B.1):

$ReLU\left( x \right)=max(0.x)$ (B.1)

ReLU can die during training the model. To overcome this problem, the Leaky ReLU is introduced which an alpha parameter in this function doesn't allow the gradient is zero when x < 0. Leaky ReLU is shown in Eq. (B.2):

$Irelu\left( x \right)= \left\{ \begin{aligned} \alpha x \\ x \end{aligned}\begin{matrix} if x\leq0 \\ if x>0 \end{matrix} \right.$ (B.2)

The SOFTMAX activation function is used in the last dense layers for multi-class classification problem as Eq. (B.3):

$SOFTMAX\left( y_{i} \right)= \frac{y_{i}}{\sum_{j} y_{j}}$ (B.3)
